# Chemobrain as a Neuroimmune Syndrome: Mechanisms, Modifiers, and Emerging Multi-Target Therapeutic Strategies

**DOI:** 10.3390/molecules31111796

**Published:** 2026-05-23

**Authors:** Federica Carnemolla, Sandeep Kumar Singh, Leonardo Ceccherini, Niccolò Taddei, Monica Bucciantini, Manuela Leri

**Affiliations:** 1 Department of Experimental and Clinical Biomedical Sciences, University of Florence, 50134 Florence, Italy; federica.carnemolla@unifi.it (F.C.); leonardo.ceccherini2@edu.unifi.it (L.C.); niccolo.taddei@unifi.it (N.T.); 2 Department of Medical Biotechnology, All India Institute of Medical Sciences (AIIMS) Nagpur, Nagpur 441108, Maharashtra, India; sandeeps.bhu@gmail.com

**Keywords:** estrogen-related pathways, chemotherapy-induced cognitive impairment (CICI), neuroinflammation, mitochondrial dysfunction, dietary phenolic subclasses

## Abstract

Chemotherapy-induced cognitive impairment (CICI), often referred to as “chemobrain,” is a common and sometimes persistent consequence of cancer treatment, characterized by deficits in memory, attention, executive function, and processing speed; it disproportionately affects older adults and women, suggesting a role for aging- and sex-related biological factors, including estrogen depletion. This work examines the potential of dietary phenolic compounds as multi-target modulators of mechanisms underlying CICI. A narrative synthesis of preclinical and clinical evidence was conducted, focusing on major phenolic subclasses (flavonoids, phenolic acids, stilbenes, lignans, and secoiridoids) and their effects on pathways implicated in chemotherapy-related neurotoxicity. The reviewed data indicate that phenolic compounds can influence redox balance, neuroinflammatory responses, mitochondrial function, synaptic plasticity, and estrogen-related signaling, with effects that appear to be structure-dependent; however, evidence remains heterogeneous and largely derived from experimental models rather than studies in humans. Overall, the current findings suggest that selected phenolic compounds could mitigate vulnerability to CICI, particularly in higher risk groups such as older individuals and women with low estrogen levels. These compounds represent promising and safe adjunctive strategies, although further well-designed clinical studies are needed to confirm their efficacy and clarify the underlying mechanisms.

## 1. Introduction

Chemotherapy-induced cognitive impairment (CICI), commonly referred to as “chemobrain,” is a significant yet often underestimated challenge among cancer survivors, particularly in women treated for breast cancer. It is characterized by deficits in memory, attention, processing speed, and executive function. Reported prevalence ranges widely (4–80%), reflecting heterogeneity in patient populations, treatment regimens, and cognitive assessment methods [1]. Initially attributed to the direct neurotoxic effects of chemotherapeutics agents, CICI is now widely recognized as a multifactorial condition involving age-related vulnerability, systemic inflammation, hormonal changes, and genetic susceptibility [2,3]. In particular, estrogen deprivation appears to exacerbate cognitive decline, highlighting the importance of hormonal context in shaping the brain’s response to chemotherapy [4,5]. At the biological level, CICI is primarily driven by neuroinflammatory processes and impaired neurogenesis. For example, chemotherapeutic agents such as cisplatin have been shown to disrupt hippocampal neurogenesis, partly through modulation of adenosine A_2_A receptor signaling [6,7]. In parallel, pro-inflammatory cytokines interact with psychosocial factors, including stress and depression, influencing cognitive trajectories [8,9].

CICI shares several molecular pathways with neurodegenerative disorders, such as neuroinflammation and synaptic dysfunction, but differs fundamentally in its etiology [10]. Unlike Alzheimer’s disease (AD), which is characterized by amyloid-β and tau pathology leading to progressive neuronal loss [11], CICI arises mainly by treatment-related and systemic factors such as cytokine-mediated neuroinflammation, blood–brain barrier disruption, and mitochondrial dysfunction, and is often at least partially reversible [12]. Notably, cognitive impairment may occur even before chemotherapy initiation, supporting a role for tumor-driven systemic inflammation [13]. Overall, CICI is better conceptualized as a multifactorial and dynamic neuroimmune syndrome shaped by the interplay between cancer biology, treatment exposure, and individual susceptibility, rather than a primary neurodegenerative or purely inflammatory condition [13,14].

Recent meta-analyses confirm the high prevalence of CICI following chemotherapy, highlighting the urgent need for greater standardization of cognitive assessment tools, in order to improve comparability across studies and populations [15,16]. Increasing attention is also being given to early evaluation and supportive interventions aimed at preserving cognitive function. Approaches such as structured physical activity programs have shown promise in enhancing cognitive resilience and maintaining functional independence among breast cancer survivors [17,18]. Collectively, these observations suggest the need for a more integrated approach to CICI management, combining systematic cognitive assessment with pharmacological, lifestyle, and supportive strategies, all of which would be tailored to the needs of vulnerable patient groups. In line with this integrative perspective, this review adopts a narrative approach based on a comprehensive literature search conducted across major scientific databases, including PubMed, Scopus, and Web of Science. Articles published up to March 2026 were retrieved using combinations of relevant keywords such as “chemotherapy-induced cognitive impairment”, “chemobrain”, “polyphenols”, “flavonoids”, “neuroinflammation”, “oxidative stress”, and “estrogen-related signaling”. Additional studies were identified through manual screening of the reference lists of selected articles. Both preclinical and clinical studies were considered, with priority given to recent and high-quality evidence addressing the molecular mechanisms underlying CICI and the potential neuroprotective role of dietary phenolic compounds. While not structured as a systematic review, this work aims to provide a comprehensive and integrative overview of the current state of knowledge.

## 2. Chemotherapy-Induced Cognitive Impairment (CICI)

### 2.1. Clinical Manifestations of CICI

CICI is a complex clinical syndrome characterized by measurable deficits across multiple cognitive domains, including attention, working memory, processing speed, and executive function, which may persist for months or even years after treatment ends [19]. As summarized in Table 1, evidence from systematic reviews and longitudinal studies indicates that a substantial proportion of breast cancer survivors experience persistent cognitive difficulties following chemotherapy. Self-reported cognitive complaints are particularly common, with approximately 36–45% within the first year after treatment [1]. Meta-analyses of 52 studies highlight the impact of assessment methods on prevalence estimates, reporting rates of 44% with self-report, 16% with screening tools, and 21–34% with neuropsychological testing, with a subset of patients showing long-term persistence of impairment [1]. Consistently, cohort studies using National Institute on Aging–Alzheimer’s Association (NIA-AA) diagnostic criteria indicate that approximately 20% of breast cancer patients develop mild cognitive impairment 6–12 months after chemotherapy, compared with 7.6% of controls [20]. More recent investigations converge on an overall prevalence of 30–50%, whereas earlier estimates ranged from 15% to as high as 75%, largely due to methodological differences [1,20,21].

Several classes of chemotherapeutic agents have been associated with CICI, giving rise to cognitive deficits that, while partly overlapping, also show drug-specific patterns affecting attention, memory, executive function, and processing speed (Table 2). Among these, anthracyclines such as doxorubicin have been consistently linked to impairments in memory and learning, largely driven by oxidative stress, mitochondrial dysfunction, dendritic damage, and disruption of the autophagy–lysosome system [22,23,24,25]. Antimetabolites, including methotrexate, are also frequently implicated, likely through effects on white matter integrity and glial function. Taxane-based therapies tend to impact attention and executive processes [26], whereas platinum compounds are more often associated with memory deficits linked to oxidative stress and mitochondrial impairment [27]. Despite these differences, these agents ultimately converge on a set of common biological pathways, such as neuroinflammation, impaired neurogenesis, mitochondrial dysfunction and white matter damage, which help explain the variability in cognitive outcomes observed among cancer survivors.

In addition to treatment-related mechanisms, modifiable lifestyle factors, particularly sleep disruption, have emerged as important contributors to CICI. Chronic sleep deprivation can promote maladaptive microglial activation, impair glymphatic clearance, and enhance neuroinflammation, thereby exacerbating cognitive decline in combination with chemotherapy- and age-related processes [30].

Given this complexity, accurate characterization and diagnosis of CICI requires a multimodal approach that integrates self-reported symptoms, cognitive testing, and advanced neuroimaging techniques, which have revealed both structural and microstructural alterations in gray and white matter regions involved in memory and executive function, including hippocampal atrophy and disrupted white matter integrity [31,32]. While some of these changes overlap with those observed in neurodegenerative conditions such as Alzheimer’s disease, CICI remains distinct in its treatment-related origin, its temporal association with chemotherapy exposure, and its potential for partial reversibility. In this context, the dynamic interplay among systemic inflammation, cancer-related factors, sleep disturbance, and therapy-induced hormonal changes defines a unique and clinically relevant neurobiological landscape underlying cognitive impairment in cancer patients.

### 2.2. Pathophysiological Mechanisms

The biological basis of CICI is not driven by a single neurotoxic mechanism but rather arises from the convergence of multiple, closely interacting processes. Oxidative stress, mitochondrial dysfunction, neuroinflammation, altered neurotransmission, epigenetic changes, estrogen depletion, and cellular senescence act together to disrupt neural homeostasis and ultimately impair cognitive function [33].

Within this complex landscape, neuroinflammation appears to play a central role, linking systemic alterations to changes in brain function [33]. At the same time, growing evidence points to the involvement of the gut–brain axis, as chemotherapy-induced dysbiosis and impairment of the intestinal barrier can sustain systemic inflammation and further influence neural processes.

Taken together, these observations support the view of CICI as a neuroinflammatory condition shaped by the continuous interplay of systemic, metabolic, and neural factors.

Importantly, these mechanisms are not independent but deeply interconnected. In particular, oxidative stress and mitochondrial dysfunction feed into each other in a self-reinforcing cycle: increased production of reactive oxygen species damages mitochondrial components, impairing energy metabolism and further promoting ROS generation. In turn, mitochondrial dysfunction triggers neuroinflammatory responses, including microglial activation and cytokine release. At the same time, persistent neuroinflammation further amplifies oxidative stress through the production of reactive species and inflammatory mediators, thereby aggravating mitochondrial damage.

Overall, these processes form a self-sustaining loop that drives neuronal dysfunction, impairs synaptic plasticity, and limits neurogenesis. Viewing these alterations as part of an integrated network is key to identifying effective multi-target therapeutic strategies capable of disrupting the pathological cascades underlying CICI.

#### 2.2.1. Oxidative Stress and Mitochondrial Dysfunction

Oxidative stress and mitochondrial dysfunction are among the most consistently implicated mechanisms in CICI. Chemotherapeutic agents such as doxorubicin increase reactive oxygen species (ROS) production while impairing antioxidant defenses, leading to mitochondrial DNA damage and activation of apoptotic pathways [22,34] while simultaneously impairing endogenous antioxidant defenses. These alterations lead to mitochondrial DNA damage, impaired electron transport chain activity, and activation of apoptotic and necroptotic pathways. Other agents, such as cisplatin, cyclophosphamide and methotrexate, directly damage mitochondrial DNA by forming adducts or interstrand crosslinks, impairing electron transport chain complexes I, II, and IV. The resulting decline in ATP production further exacerbates ROS generation and promotes both apoptotic and necroptotic cell death [22,35,36]. Together, these processes establish a self-perpetuating cycle in which mitochondrial dysfunction enhances oxidative stress, leading to sustained neuronal damage and progressive synaptic loss, impaired neurogenesis, and cognitive deficits [37]. Notably, targeting mitochondrial dynamics has emerged as a promising therapeutic strategy. In a rat model of doxorubicin-induced CICI, pharmacological inhibition of mitochondrial fission with mitochondrial division inhibitor 1 (Mdivi-1), combined with promotion of mitochondrial fusion using mitochondrial fusion promoter M1 (M1), restored cognitive performance in behavioral tasks. These interventions attenuated oxidative stress, neuroinflammation, synaptic degeneration, apoptosis, and necroptosis, alongside reduced activation of TNF-α and the receptor-interacting serine/threonine-protein kinase 1 (RIPK1), receptor-interacting protein kinase 3 (RIPK3), mixed lineage kinase domain like pseudokinase (MLKL) signaling pathway in the hippocampus [38]. Consistent with these findings, antioxidant and anti-inflammatory agents such as C-phycocyanin and rosuvastatin have shown neuroprotective effects in experimental models. These compounds preserve mitochondrial ultrastructure, restore antioxidant defence such as glutathione (GSH) and superoxide dismutase (SOD), reduce MDA and TNF-α levels, and sustain brain-derived neurotrophic factor (BDNF)/cAMP response element-binding protein (CREB)/extracellular signal-regulated kinase (ERK) signaling, thereby supporting synaptic integrity and cognitive function [39,40,41]. Collectively, these observations suggest that restoring mitochondrial function, reinforcing antioxidant defenses, and modulating neurotrophic signaling represent effective strategies to mitigate CICI.

#### 2.2.2. Neuroinflammation

Neuroinflammation is a central mechanism in CICI, with elevated pro-inflammatory cytokines linked to post-treatment cognitive deficits and supporting its role as a key driver of disease pathophysiology [33,42]. Among key mediators, IL-1β, a potent activator of NF-κB signaling, and TNF-α, which promotes N-methyl-D-aspartate (NMDA) receptor-mediated excitotoxicity while reducing neurotrophic support, including BDNF, play prominent roles in disrupting synaptic plasticity and ultimately impairing learning and memory processes [43,44,45]. Chemotherapy further amplifies inflammatory responses through activation of microglia in a pro-inflammatory phenotype (M1), characterized by the release of ROS, IL-1β, TNF-α, inducible nitric oxide synthase (iNOS), and other cytotoxic mediators. This sustained activation contributes to synaptic degeneration, dendritic spine loss, and neuronal injury [46,47]. Importantly, microglial activation is not transient but may persist over time, reinforcing neuroinflammatory signaling and prolonging cognitive vulnerability. Within this inflammatory landscape, the NLR Family Pyrin Domain Containing 3 (NLRP3) inflammasome represents a key link between chemotherapy and sustained neuroinflammation. Upon activation in microglia and astrocytes, it assembles with apoptosis-associated speck-like protein (ASC) and caspase-1, promoting the release of IL-1β and IL-18 and inducing pyroptotic cell death [48,49,50]. Pharmacological inhibition of NLRP3 reduces cytokine release and improves cognitive performance in preclinical models, supporting its therapeutic potential [51,52]. Overall, these pathways highlight neuroinflammation as a central driver of CICI and a potential therapeutic target.

#### 2.2.3. Neurotransmitter Disruption

Chemotherapy-induced alterations in neurotransmitter systems, particularly dopaminergic and cholinergic pathways, contribute significantly to cognitive dysfunction. Reduced dopamine signaling has been observed in both clinical and preclinical studies, leading to deficits in executive function and working memory [47,53]. Consistently, in preclinical models, carboplatin treatment markedly impairs dopamine release and uptake in the striatum, leading to deficits in executive control and working memory [53]. Cholinergic neurotransmission is also affected, with reduced acetylcholine availability and impaired receptor activation negatively compromising synaptic plasticity and memory processes. In line with these findings, clinical trials using the acetylcholinesterase inhibitor donepezil, which increases acetylcholine levels by preventing its degradation, have reported modest improvements in attention and verbal memory in cancer survivors [54]. Overall, disruption of dopaminergic and cholinergic neurotransmission contributes to dysfunction of prefrontal–striatal and hippocampal networks involved in executive control and memory consolidation, highlighting neurotransmitter imbalance as a relevant component of CICI pathogenesis and a potential target within multi-modal therapeutic strategies [54,55].

#### 2.2.4. Epigenetic Modifications

Epigenetic alterations have emerged as important contributors to CICI. These modifications, including DNA methylation and histone acetylation, regulate gene expression without altering the DNA sequence and play essential roles in neural plasticity, synaptic function, and brain homeostasis.

Chemotherapeutic agents can disrupt normal DNA methylation patterns, leading to dysregulation of genes involved in neuroplasticity and cognition [56,57].

Reduced expression of neurotrophic factors such as BDNF and Sry-related HMG-box 10 (SOX10) has been observed following treatment with agents such as cisplatin or methotrexate [58,59]. Although the direct involvement of promoter hypermethylation remains to be fully established, the downregulation of these genes has been associated with impaired synaptic plasticity and reduced cognitive resilience in cancer survivors. Moreover, altered methylation of genes related to glial activation and cytokine signaling may contribute to the persistence of neuroinflammatory responses [42].

Beyond DNA methylation, chemotherapy also affects histone acetylation dynamics. Agents such as doxorubicin, cyclophosphamide, and 5-fluorouracil disrupt the balance between histone acetyltransferases (HATs), which promote gene transcription by loosening chromatin structure, and histone deacetylases (HDACs), which promote chromatin condensation and transcriptional repression. This imbalance often favors hypoacetylation leading to the silencing of neuroprotective genes [60,61]. Functionally, these epigenetic alterations impair long-term potentiation (LTP), reduce dendritic spine density, and exacerbate oxidative stress and mitochondrial dysfunction, ultimately contributing to cognitive decline [61,62].

#### 2.2.5. Telomere Shortening and Cellular Senescence

Chemotherapy has been associated with features of accelerated biological aging, including telomere shortening and accumulation of senescent cells. These changes promote a senescence-associated secretory phenotype (SASP), characterized by the release of pro-inflammatory mediators that amplify neuroinflammation and oxidative stress [63], linking cellular senescence to the clinical manifestations of CICI.

Within this framework, adenosine A_2_A receptor (A_2_AR) signaling emerges as a key modulator that, while not directly driving telomere shortening, amplifies its downstream consequences by promoting neuroinflammation, oxidative stress, and microglial activation, thereby reinforcing SASP and neuronal dysfunction. Under chemotherapy-induced stress, increased extracellular adenosine leads to A_2_AR overactivation, linking mitochondrial impairment, synaptic dysfunction, and reduced neurogenesis, hallmarks of accelerated brain aging and cognitive decline.

Consistently, preclinical studies have shown that cisplatin increases hippocampal A_2_AR expression, whereas pharmacological blockade of A_2_AR preserves neurogenesis, dendritic integrity, and cognitive performance while reducing anxiety-like behavior [7].

Collectively, these findings suggest that targeting interconnected processes such as neuroinflammation, mitochondrial dysfunction, and cellular senescence, including modulation of A_2_AR signaling, may represent a promising strategy to preserve cognitive function.

#### 2.2.6. Estrogen’s Involvement

Cancer therapies targeting hormone-sensitive malignancies such as estrogen receptor-positive (ER+) breast cancer frequently cause significant estrogen depletion. This occurs through chemotherapy-induced ovarian failure or endocrine therapies such as aromatase inhibitors and GnRH analogs and may be further reinforced by surgical oophorectomy [64,65,66].

Chemotherapy regimens containing cyclophosphamide, doxorubicin, and paclitaxel can rapidly reduce circulating estradiol (E2) levels to a postmenopausal range. This reduction is often accompanied by decreased ovarian reserve markers, including anti-Müllerian hormone (AMH) and inhibin B, indicating persistent ovarian dysfunction. Given its central role in cognitive function, estrogen depletion contributes to CICI by converging on several of the pathogenic mechanisms described above. In particular, reduced estrogen signaling exacerbates core CICI-related pathways, including deficits in synaptic plasticity and neurogenesis, increased mitochondrial dysfunction, and enhanced neuroinflammatory responses, thereby increasing vulnerability to cognitive impairment [67,68,69,70].

At the molecular level, estrogen exerts neuroprotective effects mainly via ERα and ERβ receptors, highly expressed in cognition-related brain regions. Their activation promotes dendritic spine formation, synaptic plasticity, cholinergic and glutamatergic neurotransmission. Estrogen also enhances antioxidant defenses, upregulating enzymes such as SOD and glutathione peroxidase (GPx), and suppresses neuroinflammation by inhibiting NF-κB signaling and reducing pro-inflammatory cytokines such as IL-1β and TNF-α. Experimental studies consistently show that estrogen deprivation impairs memory and synaptic plasticity, effects that can be reversed by estradiol supplementation. Clinically, reduced estrogen levels are associated with poorer performance in attention, memory, and executive function [70].

Endocrine aging further complicates this scenario. The decline in estradiol during menopause is associated with changes in mood, sleep, and cognition and contributes to neuropsychiatric and neurodegenerative conditions. Brain aging and endocrine changes act synergistically, converging on mechanisms such as oxidative stress, mitochondrial dysfunction, and neuroinflammation, which may further potentiate chemotherapy-induced toxicity.

The major pathophysiological mechanisms involved in CICI, together with their cognitive consequences and potential therapeutic targets, are summarized in Table 3.

## 3. Risk Factors and Sex Differences in Cognitive Vulnerability

CICI is a multifactorial condition influenced by a complex interplay of patient-related, treatment-related, and biological factors. In addition to age, genetic susceptibility, comorbidities, psychosocial stress, and lifestyle variables, sex- and gender-related differences have emerged as important modulators of both susceptibility and clinical expression of cognitive impairment [71,72].

Clinical evidence consistently indicates that women are disproportionately affected by CICI, particularly in settings characterized by estrogen deprivation, such as menopause or chemotherapy-induced amenorrhea (CIA).

Depending on treatment regimens, CIA occurs in approximately 20–80% of premenopausal breast cancer survivors and has been associated with altered recruitment of working memory networks, particularly in the prefrontal cortex. Increased neural activation in these regions has been linked to slower processing speed, suggesting reduced neural efficiency rather than effective compensatory mechanisms [73].

A key contributor to this vulnerability is the neuroprotective role of estrogen. Beyond its endocrine functions, estrogen modulates multiple pathways implicated in CICI pathophysiology, including neurotransmitter systems, (cholinergic, serotonergic, and dopaminergic), mitochondrial function, oxidative balance, and neuroinflammatory signaling. Experimental and clinical studies have shown that estrogen supports mitochondrial efficiency and cerebral metabolism and limits pathological processes such as β-amyloid toxicity and tau hyperphosphorylation, particularly in brain regions critical for cognition, including the hippocampus and prefrontal cortex [74,75]. At the synaptic level, estrogen facilitates acetylcholine synthesis and release, thereby sustaining cholinergic transmission in memory-related circuits [76], and promotes activity-dependent plasticity through upregulation of BDNF [77,78,79]. In parallel, estrogen dampens oxidative stress and neuroinflammation by reducing reactive oxygen species and downregulating pro-inflammatory cytokine signaling [80]. The timing of estrogen exposure appears critical, with evidence supporting a “critical window” during which hormone replacement may preserve cognitive function, whereas delayed intervention shows limited benefit [81].

**Figure 1:**
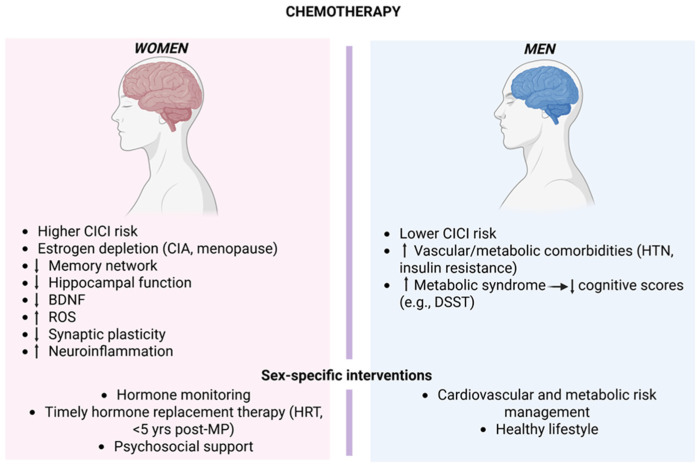
Sex-specific differences in chemotherapy-induced cognitive impairment. Women show increased vulnerability, particularly in estrogen-deficient conditions, while cognitive decline in men is more closely linked to vascular and metabolic factors. Potential sex-specific interventions are indicated. ↑ indicates increase; ↓ indicates decrease.

Cognitive vulnerability in women is further influenced by psychosocial stressors and brain changes associated with abrupt estrogen withdrawal, including alterations in hippocampal connectivity. In men, by contrast, cognitive decline associated with chemotherapy appears to be driven more prominently by vascular and metabolic factors than by hormonal changes. Population-based analyses from the National Health and Nutrition Examination Survey (NHANES) indicate that the accumulation of metabolic syndrome features is associated with progressively poorer performance on the Digit Symbol Substitution Test in adults aged 60 years and older, with increasingly negative β-coefficients as metabolic burden rises [82]. These observations point to cardiovascular and metabolic health as key modifiers of cognitive resilience in male cancer survivors. Importantly, sex differences in CICI extend beyond endocrine mechanisms. Evidence from long-term survivors of acute lymphoblastic leukemia (ALL) treated with chemotherapy shows that female survivors more frequently report difficulties in memory and emotion regulation compared with males, suggesting an intrinsic sex-related susceptibility to neurocognitive sequelae [83]. At the same time, distinct treatment-related patterns have been observed: methotrexate affects both sexes, whereas dexamethasone appears to increase memory deficits primarily in males. Similarly, neurological comorbidities are more strongly associated with cognitive impairment in males, while endocrine conditions are more closely linked to deficits in females. Functional neuroimaging further supports sex-specific differences in brain organization, with female survivors showing reduced neural efficiency during working memory tasks, despite comparable performance [84]. Overall, cognitive vulnerability after chemotherapy appears to be influenced by sex-specific biological, hormonal, and clinical factors, highlighting the importance of tailored prevention and management strategies. These sex-related differences and the corresponding potential intervention strategies are summarized in Figure 1. In women, particular attention to hormonal status and the potential role of timely hormone-based interventions may be beneficial, while in men, managing vascular and metabolic risk factors may be more relevant. Together, these observations support the need for personalized, sex-informed approaches to preserving cognitive function in cancer survivors [85,86].

## 4. Neuroprotective Strategies and Interventions

### 4.1. Pharmacological Treatments

Despite increasing awareness of CICI, no pharmacological treatments have yet been approved by the U.S. Food and Drug Administration (FDA) for its prevention or management. Several agents have nonetheless been explored for their potential to alleviate cognitive deficits, particularly in attention, memory, and executive function, with overall mixed and modest results.

Modafinil, a wakefulness-promoting agent approved for narcolepsy and sleep disorders, has shown some promise in improving cognitive performance in breast cancer survivors at a dose of 200 mg/day. Randomized trials report modest and selective improvements in attention and vigilance; however, its clinical utility is limited by adverse effects such as headache, insomnia, nervousness, and nausea, as well as concerns regarding long-term tolerability and potential misuse [87].

Cholinergic enhancement has also been investigated. Donepezil, an acetylcholinesterase inhibitor widely used in Alzheimer’s disease, demonstrated improvements in verbal memory and executive function in a 24-week phase II trial in breast cancer survivors [88]. Nonetheless, its use may be constrained by gastrointestinal side effects, bradycardia, and sleep disturbances.

Central nervous system stimulants such as methylphenidate have been primarily evaluated for cancer-related fatigue, with only limited cognitive benefits observed [89]. Adverse effects, including anxiety, agitation, appetite suppression, and cardiovascular concerns, together with the risk of dependence, frequently restrict their clinical applicability.

Memantine, an NMDA receptor antagonist, has been shown to reduce excitotoxicity and delay cognitive decline in patients undergoing whole-brain radiotherapy, as demonstrated in a randomized, double-blind, placebo-controlled trial [90]. Although this evidence derives from radiation-induced cognitive impairment rather than chemotherapy-related toxicity, the underlying neuroprotective mechanisms suggest potential relevance for broader cancer treatment contexts, including CICI. However, direct clinical evidence supporting its use specifically in chemobrain remains limited.

Overall, while these pharmacological approaches may offer symptomatic relief in selected patients, their clinical benefits are generally modest and highly variable. Moreover, side-effect profiles and common comorbidities in cancer populations necessitate careful risk–benefit evaluation. Pharmacologic strategies are therefore best considered within a broader, multimodal framework that includes non-pharmacological interventions such as cognitive rehabilitation, physical exercise, and psychosocial support. Large-scale, well-designed trials remain essential to establish efficacy, optimize dosing, and assess long-term safety in diverse cancer survivor populations.

### 4.2. Non-Pharmacological Treatments

In the absence of approved pharmacological therapies, non-pharmacological approaches have gained increasing relevance in the management of CICI. Their value lies not only in their favorable safety profile but also in their ability to act on multiple, interconnected mechanisms underlying cognitive impairment.

Among these, lifestyle interventions remain the most consistently supported. Aerobic and resistance exercise, structured cognitive training, and stress-reduction strategies have shown measurable benefits on both subjective and objective cognitive outcomes, likely through improvements in neurotrophic signaling, cerebral perfusion, and systemic inflammation [91,92].

Alongside lifestyle approaches, nutritional strategies are emerging as an important complementary component. In this context, growing attention has been directed toward the gut–brain axis as a key interface between systemic and neural processes in CICI. Chemotherapy can disrupt gut microbiota composition, increasing intestinal permeability and promoting the translocation of pro-inflammatory mediators into the circulation [93]. This, in turn, sustains chronic low-grade inflammation and reinforces neuroinflammatory pathways.

At a mechanistic level, microbial-derived metabolites play a central role. Short-chain fatty acids (SCFAs), particularly butyrate, support blood–brain barrier integrity and help regulate microglial activity, whereas their depletion has been linked to increased neuroinflammation and cognitive dysfunction. In parallel, alterations in the tryptophan–kynurenine pathway may contribute to neurotransmitter imbalance and excitotoxicity.

Within this framework, several dietary compounds have been explored for their potential to mitigate CICI, including polyphenols, carotenoids, and omega-3 fatty acids [94]. Omega-3 fatty acids, particularly eicosapentaenoic acid (EPA) and docosahexaenoic acid (DHA), have shown protective effects in preclinical models, although clinical evidence remains inconsistent [95,96,97]. Medium-chain fatty acids, such as lauric acid, may also contribute by providing alternative energy substrates and modulating inflammatory pathways, although their role in CICI is still under investigation [98,99].

Among these approaches, dietary polyphenols have attracted particular interest. Beyond their well-known antioxidant properties, these compounds can modulate key biological processes involved in CICI, including inflammation, mitochondrial function, and synaptic plasticity. Importantly, polyphenols also interact with the gut microbiota, shaping its composition and metabolic activity. These bidirectional interactions influence their bioavailability and suggest that part of their neuroprotective effects may be mediated indirectly through microbiota-dependent mechanisms. Consistent with this view, plant-derived compounds such as Gastrodia Elata and flavonoids like isovitexin have been shown to modulate oxidative stress, ferroptosis, and gut-related pathways in experimental models [100,101].

Despite these promising observations, several limitations must be considered. The intake of polyphenols varies widely across dietary patterns, and their bioavailability is often limited by rapid metabolism and clearance. As a result, their biological effects are largely mediated by circulating metabolites rather than the parent compounds, and the concentrations achieved through diet alone may not always reproduce those observed in experimental settings. These aspects highlight the importance of considering both dietary patterns and optimized delivery strategies [96].

Overall, current evidence supports a multimodal approach to CICI management, combining lifestyle interventions with anti-inflammatory dietary patterns. Meanwhile specific Mediterranean (MD) and Mediterranean-DASH Intervention for Neurodegenerative Delay (MIND) diets are likely to provide more consistent and clinically relevant benefits than isolated supplementation [96].

#### 4.2.1. Phenolic Compounds and Related Phytochemicals in the Prevention of Chemobrain

Among the nutritional strategies discussed above, dietary polyphenols have attracted particular interest due to their broad biological activity and potential to modulate key mechanisms involved in CICI. Many of the molecular pathways implicated in chemotherapy-induced cognitive impairment, including oxidative stress, neuroinflammation, mitochondrial dysfunction, and estrogen-related signaling, are directly influenced by polyphenols, supporting their role as modulators of chemotherapy-related neurotoxicity.

Rather than acting through isolated mechanisms, polyphenols exert pleiotropic effects by converging on key stress-responsive molecular hubs, including 5′ AMP-activated protein kinase (AMPK), Sirtuin1 (SIRT1), PGC-1α, NF-κB, cyclooxygenase enzymes, and autophagy-related pathways, that collectively govern redox balance, energy metabolism, inflammatory responses, and synaptic integrity during chemotherapeutic stress [96]. This systems-level mode of action is especially relevant in CICI, a condition characterized by the simultaneous disruption of multiple biological processes. This group encompasses structurally diverse molecules, including flavonoids (e.g., genistein), stilbenes (e.g., resveratrol), curcuminoids (e.g., curcumin), and olive oil–derived secoiridoids and simple phenols such as oleuropein aglycone (OleA) and hydroxytyrosol (HT). Their biological activity is strongly influenced by their chemical structure, including hydroxylation patterns, degree of conjugation, and glycosylation, which affect their antioxidant capacity, receptor interactions, and bioavailability.

The number and position of hydroxyl groups on aromatic rings play a key role in determining antioxidant capacity and radical scavenging activity, with higher hydroxylation typically enhancing reactivity and metal-chelating properties. Likewise, structural elements such as conjugation and molecular planarity, particularly the presence of an unsaturated bond coupled with a carbonyl group, facilitate interactions with proteins and signaling pathways, thereby influencing anti-inflammatory and neuroprotective effects. Modifications such as glycosylation, methylation, and polymerization further affect solubility, stability, and bioavailability, often altering target affinity and cellular uptake. These structural features also enable polyphenols to interact dynamically with proteins through hydrogen bonding and other non-covalent interactions, supporting their ability to modulate key pathways involved in CICI. Overall, these structure–activity relationships help explain why different subclasses of polyphenols show variable efficacy in targeting oxidative stress, neuroinflammation, and mitochondrial dysfunction [95].

Within this framework, polyphenols may contribute to brain resilience during chemotherapy by modulating shared molecular pathways. Supporting this, preclinical studies have shown that curcumin improves cisplatin-treated mice’s hippocampal-dependent cognitive performance by activating AMPK–c-Jun N-terminal kinase (JNK) signaling, enhancing autophagy, and promoting neurogenesis and synaptogenesis, while normalizing apoptosis-related pathways [102]. Evidence from human studies further supports these findings. A systematic review and meta-analysis of randomized controlled trials reported that curcumin supplementation at approximately 0.8 g/day for at least 24 weeks significantly improved global cognitive performance, with more pronounced effects in older individuals and Asian populations [103]. Similarly, in older adults without dementia, long-term supplementation with a bioavailable curcumin formulation (Theracurmin^®^) was associated with improvements in verbal and visual memory, attention, and reduced amyloid and tau PET binding in brain regions relevant to cognition [104]. In a double-blind, placebo-controlled randomized trial, eighty cancer patients on standard chemotherapy regimens were randomly assigned to receive curcumin as adjuvant therapy (500 mg every 12 h for 9 weeks) showed improvements in clinical symptoms [105]. Comparable patterns emerge for other polyphenols. In models of paclitaxel-induced neurotoxicity, resveratrol improved spatial learning, reduced neuronal apoptosis and oxidative stress, and promoted a shift toward an anti-inflammatory microglial phenotype through activation of the SIRT1/PGC-1α axis, a key regulator of mitochondrial function and cellular energy metabolism [92].

Genistein, acting on overlapping stress-response pathways, was evaluated in a year-long clinical trial in patients with prodromal Alzheimer’s disease, where daily supplementation (120 mg) improved verbal learning, and delayed recall on the Rey Complex Figure Test, while attenuating amyloid-β accumulation in the anterior cingulate cortex, suggesting sustained modulation of neurodegeneration-related signaling networks [106].

Olive oil phenolics further illustrate this convergent molecular profile. OleA and HT modulate neuroinflammatory signaling by inhibiting cyclooxygenase (COX)-1/2 activity, regulating microglial triggering receptor expressed on myeloid cells 2 (TREM2) signaling, reducing pro-inflammatory cytokine release (including IL-6, IL-8, interferon gamma-induced protein 10 (IP-10), and regulated on activation, normal T-cell expressed and secreted (RANTES)). At the same time, they enhance autophagy and activate the AMPK–SIRT1–PGC-1α axis, thereby improving mitochondrial efficiency and reducing reactive oxygen species in neuronal models [107,108]. Consistent with these molecular effects, preclinical studies in CICI mouse models show that EVOO supplementation suppresses hippocampal inflammation (e.g., TNF-α and IL-1β), attenuates oxidative stress, enhances autophagy markers such as Microtubule-associated protein 1A/1B-light chain 3-II (LC3-II) and Beclin-1, downregulates COX-2, and improves performance in spatial memory and recognition tasks. Despite their chemical heterogeneity, these compounds consistently target a core network of pathways involved in cellular stress adaptation. This convergence provides a strong mechanistic rationale for their use as low-toxicity modulators of brain resilience in the context of chemotherapy. However, challenges related to bioavailability, interindividual variability, and limited clinical validation remain significant barriers to translation.

#### 4.2.2. Phenolic Compounds as Phytoestrogens

Several phenolic compounds exert phytoestrogenic activity by structurally and functionally mimicking E2 and binding estrogen receptors ERα and ERβ, generally exhibiting higher affinity for ERβ [109,110,111]. Among well-characterized examples, genistein and daidzein preferentially activate ERβ, whereas coumestrol binds robustly to both ER subtypes, demonstrating up to 185% of estradiol’s binding affinity [31,111]. In addition to classical receptor-mediated actions, certain extra virgin olive oil (EVOO)-derived phenolics, such as OleA and HT, also modulate estrogen signaling through non-classical pathways; in particular, they act as inverse agonists on the G-protein-coupled estrogen receptor (GPER) in GPER-positive, ER-negative cells, through activation of the mitogen-activated protein kinase/extracellular signal-regulated kinase (MAPK/ERK) signaling cascade and influencing apoptosis processes [112]. Furthermore, HT derivatives such as HT-acetate have been shown to enhance cognitive performance and upregulate hippocampal ERβ expression in Alzheimer’s disease models, underscoring ERβ’s neuroprotective role [113]. These compounds also engage non-genomic signaling pathways, including PI3K/Akt, MAPK/ERK1/2, and protein kinase C (PKC), leading to CREB activation, a key regulator of synaptic plasticity, neurogenesis, and cognitive resilience [111]. Their antioxidant capacity is mediated in part through activation of the Nrf2/ARE pathway, which promotes the expression of cytoprotective enzymes such as SOD, GPx, glutathione S-transferase (GST), and heme oxygenase-1 (HO-1), thereby mitigating oxidative stress [114,115]. Concurrently, these compounds suppress neuroinflammation by inhibiting NF-κB signaling, reducing the expression of COX-2, iNOS, and pro-inflammatory cytokines including IL-1β and TNF-α [116]. Mitochondrial protection represents an additional key mechanism. For example, OleA has been shown to preserve mitochondrial integrity by inhibiting the fission protein dynamin-related protein 1 (Drp1) in activated microglia, thereby reducing ROS production and dampening inflammatory responses [117]. Epigenetically, compounds such as genistein modulate gene expression programs involved in neuroprotection, synaptic function, and metabolic homeostasis through effects on histone acetylation, peroxisome proliferator-activated receptors (PPARs), and DNA methylation of genes including ESR1 (ERα) and tumor suppressors like breast cancer gene 1 (BRCA1) [118]. Emerging clinical evidence supports the translational relevance of these mechanisms. Trials such as MICOIL (Management of Mild Cognitive Impairment Patients with Extra Virgin Olive Oil) and MedLey have demonstrated that EVOO rich in phenolic compounds improves cognitive performance, including memory, executive function, and processing speed, in older adults, including Apolipoprotein E4 (APOE4) carriers [119]. Additional compounds such as mangiferin and morin further contribute to neuroprotection by limiting excitotoxicity through restoration of mitochondrial function, reduction of ROS, inhibition calpain activation, and downregulating apoptotic proteins such as Bax and apoptosis-inducing factor (AIF), thus preventing neuronal death under ischemic or glutamate-induced stress [120]. Although these studies are not specific to CICI, they provide important proofs-of-concept for the neuroprotective potential of dietary phenolics in conditions characterized by hormonal decline and neuroinflammation.

Overall, phenolic compounds, including phytoestrogens, appear to support brain health by acting on multiple interconnected pathways, including estrogen signaling, oxidative balance, inflammation, mitochondrial function, and epigenetic regulation. This broad activity makes them particularly relevant in conditions of estrogen deficiency. In this context, polyphenol-rich dietary strategies may offer a valuable complementary approach to mitigate cognitive decline in CICI. Key compounds and their mechanisms are summarized in Table 4. Nevertheless, further well-designed clinical studies are needed to better define their therapeutic potential, particularly through the integration of pharmacokinetic, biomarker, and cognitive outcomes.

## 5. Translational Potential and Clinical Implications

Dietary phenolic compounds are increasingly recognized as promising, low-risk strategies to help mitigate cognitive impairment in cancer survivors. Evidence from both preclinical and clinical studies suggests that phenolic-rich diets may support brain function by acting on multiple interconnected pathways, including oxidative stress, neuroinflammation, mitochondrial function, and epigenetic regulation. A systematic review and meta-analysis in healthy adults reported improvements in memory, attention, and executive function following supplementation with flavonoids and catechins [121,122]. Although direct evidence in oncology populations remains limited, observational studies indicate that diets rich in phenolic compounds are associated with better cognitive outcomes in breast cancer survivors [123].

These findings point toward the potential value of integrating phenolic-rich dietary approaches into survivorship care, particularly for aging women who may be more vulnerable to CICI due to estrogen depletion and age-related neurobiological changes. Clinical studies using green tea extracts, such as Polyphenon E, have also demonstrated safety and feasibility in hormone-sensitive cancers, further supporting their translational relevance [124].

Importantly, the effects of phenolic compounds may be enhanced when combined with other interventions. Approaches targeting inflammation, mitochondrial function, and key signaling pathways such as MAPK/ERK may act synergistically with dietary phenolics. Likewise, lifestyle strategies including physical activity, cognitive training, and nutritional education can strengthen their impact within a broader, multimodal framework.

At the same time, interindividual variability remains an important consideration. Differences in gut microbiota composition can influence the metabolism and bioavailability of phenolic compounds, ultimately shaping their effects on brain function. Future studies should therefore aim to better define optimal compounds, dosing strategies, and timing, while incorporating microbiome-based approaches to support more personalized interventions.

Despite encouraging findings, translating these results into clinical practice remains challenging. Key limitations include low bioavailability, variability in metabolism, lack of standardized dosing, and the limited number of large, well-controlled trials in cancer populations. Addressing these issues will be essential to fully realize the potential of polyphenols as part of clinical strategies to preserve cognitive function in cancer survivors.

## 6. Future Perspectives

Overall, current evidence supports a dual-pathology model in which estrogen deprivation interacts with chemotherapy-induced oxidative stress and neuroinflammation to drive the development of CICI. Moving forward, research should shift from largely descriptive approaches toward mechanistically informed, biomarker-driven clinical studies targeting these converging pathways.

In this context, estrogen-based neuroprotection represents a promising avenue, particularly through strategies that optimize timing and mode of intervention. Selective estrogen receptor modulators (SERMs), especially ERβ-selective agonists, may offer neurotrophic, antioxidant, and anti-inflammatory benefits while minimizing oncological risks. At the same time, targeting neuroinflammation—through modulation of microglial activation and cytokine signaling (e.g., IL-1β, IL-6, TNF-α)—remains a key priority for future translational and early-phase clinical studies.

Addressing oxidative stress and mitochondrial dysfunction is equally important. Mitochondria-targeted antioxidants and modulators of pathways such as MAPK/ERK may help preserve neuronal integrity under chemotherapeutic stress. Alongside these approaches, nutritional and lifestyle interventions are increasingly recognized as essential components of prevention strategies. In particular, phenolic compounds emerge as low-toxicity, multi-target modulators capable of influencing redox balance, inflammation, and metabolic resilience.

However, their clinical translation remains limited, reflecting pharmacokinetic constraints, methodological variability, and the intrinsic complexity of CICI, a heterogeneous condition shaped by treatment-related toxicity, systemic inflammation, genetic susceptibility, and psychosocial factors [98]. Additional challenges include defining optimal compound selection, dosing, formulation, and treatment timing, as well as establishing long-term efficacy. Interindividual variability, particularly differences in gut microbiota composition, further highlights the need for personalized approaches.

Ultimately, the integration of pharmacological, nutritional, and lifestyle-based strategies within a multimodal framework is likely to provide the greatest benefit. Advancing this integrative approach will be essential for developing effective interventions to preserve cognitive function and improve quality of life in cancer survivors, particularly in aging women with hormone-sensitive cancers.

## 7. Conclusions

CICI represents a significant and increasingly recognized challenge in cancer survivorship, disproportionately affecting women, particularly at older ages. This increased vulnerability reflects the combined effects of estrogen depletion and age-related neurobiological changes, including impaired synaptic plasticity, reduced hippocampal neurogenesis, mitochondrial dysfunction, and chronic low-grade inflammation.

Despite its prevalence, no pharmacological treatments have been approved to date, and available interventions show variable efficacy. This gap highlights the need for safe, accessible, and biologically grounded preventive strategies. In this context, dietary phenolic compounds, including curcumin, resveratrol, catechins and phenols with phytoestrogenic activities, have gained increasing attention for their ability to modulate key pathways underlying CICI. Beyond their antioxidant properties, these compounds can simultaneously influence interconnected processes, including oxidative stress, neuroinflammation, mitochondrial dysfunction, synaptic plasticity, and neurotrophic signaling. Their ability to converge on shared stress-responsive molecular networks may help explain the consistency of the neuroprotective effects observed across several experimental models. However, despite these encouraging findings, their clinical translation remains limited by issues related to bioavailability, interindividual variability, and the lack of large, well-controlled studies in cancer populations.

Moving forward, the most effective approach will likely be multimodal and personalized, combining nutritional strategies, physical activity, cognitive engagement, and management of hormonal and inflammatory risk factors. Progress will depend on well-designed, longitudinal studies capable of validating these approaches. Ultimately, such advances are essential to ensure that cancer survivors not only live longer but also preserve cognitive health and quality of life.

## 8. Limitations

Despite substantial progress, important limitations continue to shape the current understanding of CICI. Clinical studies remain highly heterogeneous, encompassing different cancer types, treatment regimens, cognitive assessment tools, and follow-up durations, which complicates cross-study comparisons and contributes to variability in reported outcomes. In addition, self-reported cognitive symptoms do not always align with objective neuropsychological measures, reflecting the complex and multidimensional nature of the condition.

Much of the current mechanistic understanding derives from preclinical models, which have been instrumental in identifying key pathways such as oxidative stress, mitochondrial dysfunction, neuroinflammation, impaired neurogenesis, and estrogen-related signaling. However, these models do not fully capture the complexity of human aging, hormonal transitions, and multimodal cancer therapies. A further limitation is the imbalance in sex representation, as many studies rely predominantly on male animals, while female models are often restricted to estrogen-deprivation paradigms. This limits the interpretation of sex-specific effects, despite the higher prevalence of CICI in women.

From a translational perspective, several challenges remain. Clinical studies are often limited by small sample sizes, short follow-up periods, and insufficient characterization of factors influencing cognitive outcomes, including mood, sleep, metabolic status, and endocrine dynamics. In particular, the limited assessment of hormonal trajectories hampers a comprehensive understanding of how estrogen depletion interacts with chemotherapy-induced neurotoxicity.

Although phenolic compounds consistently show neuroprotective effects in experimental models, clinical evidence in cancer populations remains limited. A major barrier to their application is low bioavailability, as many polyphenols are poorly soluble, unstable in the gastrointestinal environment, or rapidly metabolized before reaching target tissues. These constraints are closely linked to their structural features, including hydroxylation patterns and molecular stability [95]. Moreover, interindividual variability, particularly differences in metabolism and gut microbiota composition, may further influence their biological effects.

Another important limitation concerns the gap between experimental and dietary exposure. While polyphenol-rich foods are widely consumed, the concentrations required to reproduce pharmacological effects observed in preclinical models may not always be achievable through diet alone. This highlights the need to better define the relationship between dietary intake, bioavailability, and biological activity.

To address these challenges, both dietary and pharmacological strategies are being explored. Increasing the diversity and regular intake of polyphenol-rich foods may support sustained exposure and potential synergistic effects, while food preparation methods and co-consumption with dietary fats may enhance absorption. In parallel, advanced delivery systems, including liposomes and nanoformulations, have shown promise in improving stability, bioavailability, and targeted delivery of compounds such as curcumin, resveratrol, quercetin, and catechins. However, a universally effective strategy applicable across different polyphenol classes has yet to be established, highlighting the need for further research [95].

Finally, the lack of consensus on optimal dosing, formulation, and treatment duration, together with variability in study design, further complicates clinical translation. Addressing these gaps will require integrative and longitudinal research approaches combining standardized cognitive assessments, mechanistic biomarkers, microbiome profiling, and balanced sex representation. Such efforts are essential to clarify the therapeutic potential of phenolic compounds and support their evidence-based integration into survivorship care.

## Figures and Tables

**Table 1:** Estimated prevalence of CICI among breast cancer survivors by assessment method. Summary of prevalence rates across self-report, screening tools, neuropsychological testing, and NIA-AA criteria, with corresponding post-chemotherapy timeframes.

Assessment Method	Estimated Prevalence	Timeframe
Self-reported symptoms [1]	~36–45%	6–12 months post-chemotherapy
Brief cognitive screening tools [1,20]	~16%	Variable
Objective neuropsychological assessment [1,21]	~21–34%	Up to several years post-chemotherapy
NIA-AA defined mild cognitive impairment [20]	~20% (vs. ~7.6% controls)	6–12 months post-chemotherapy

**Table 2:** Chemotherapeutic agents associated with CICI: mechanisms and cognitive profiles.

Chemotherapeutic Agents	Primary Mechanisms	Cognitive Domains Impaired
Anthracyclines (Doxorubicin) [22,23,24,25,28,29]	- Oxidative stress - Systemic inflammation - Impaired autophagy–lysosome pathway - Reduced neuronal plasticity - Mitochondrial dysfunction - Apoptosis induction - Decreased neurogenesis	Memory, learning, processing speed
Antimetabolites (Methotrexate, 5-Fluorouracil) [27,29]	- Inhibition of DNA/RNA synthesis - Oligodendrocyte toxicity and demyelination - Inflammation - Microglia activation - Hippocampal neurogenesis suppression	Working memory, executive function, attention
Platinum compounds (Cisplatin, Oxaliplatin) [27,29]	- DNA damage to oligodendrocytes and impaired myelination - Mitochondrial dysfunction - Oxidative stress - Gut–liver axis dysregulation - Inflammation - Loss of microtubule stabilization	Memory, learning, executive function
Taxanes (Paclitaxel, Docetaxel) [26,29]	- Microtubule stabilization - Neuroinflammation	Attention, executive function, processing speed

**Table 3:** Pathophysiological mechanisms underlying CICI. Summary of major biological mechanisms contributing to CICI, including their molecular basis, cognitive consequences, and potential therapeutic targets. ↑ indicates increase/upregulation; ↓ indicates decrease/downregulation.

Mechanism	Key Molecular Events	Cognitive Consequences	Potential Interventions
Oxidative Stress and Mitochondrial dysfunction [22,36]	↑ ROS (O_2_•^−^, H_2_O_2_), ↓ antioxidants (GSH, SOD, catalase); mitochondrial DNA damage; impaired ETC (Complexes I–IV); mPTP opening; ↑ Bax/Bcl-2 ratio; cytochrome c release; activation of apoptosis and necroptosis	Synaptic loss, reduced dendritic arborization, impaired neurogenesis, memory and spatial learning deficits [37]	Mitochondrial modulators (e.g., Mdivi-1), antioxidants (C-phycocyanin, rosuvastatin), BDNF/CREB/ERK pathway activators [39,40,41]
Neuroinflammation [32,42]	↑ IL-1β, IL-18, TNF-α; NF-κB activation; microglial M1 polarization; ROS, iNOS release; ↑ NLRP3 inflammasome assembly, ↑ pyroptosis; ↑ TREM2 [48,50]	Disrupted LTP, excitotoxicity, reduced BDNF levels, impaired executive function and memory [46,47]	NF-κB inhibitors, NLRP3 inflammasome blockers, anti-inflammatory agents [44,46,70]
Neurotransmitter Disruption [54,55]	↓ Dopaminergic and cholinergic signaling; ↓ acetylcholine; impaired nicotinic/muscarinic receptor activation; synaptic dysfunction [47,53]	Executive dysfunction, attention deficits, memory consolidation impairment	Cholinesterase inhibitors (e.g., donepezil), dopaminergic enhancers, neurotransmitter rebalancing agents
Epigenetic Modifications [56,57,60,61]	↑ DNA methylation (e.g., BDNF, NRG1, GDNF gene silencing); ↓ histone acetylation; imbalance of HAT/HDAC activity; impaired gene expression for synaptic, glial, and neurogenic functions	Impaired LTP, synaptic dysfunction, reduced neurogenesis and myelination, cognitive decline	HDAC inhibitors, DNA demethylating agents, epigenetic modulators

**Table 4:** Polyphenols and CICI: mechanisms and cognitive effects. This table summarizes key dietary polyphenols and their mechanisms of action in CICI, highlighting their effects on estrogen signaling, oxidative stress, neuroinflammation, mitochondrial function, and the specific CICI-related pathways they target.

Phenolic Compounds/Source	Model/Study Type	Estrogen Receptor Activity	Key Molecular Pathways	Neurobiological & Cognitive Outcomes	Relevance to Menopause & CICI
Curcumin [102,103,104]	Cisplatin-treated mice; RCTs; meta-analysis	Indirect ER modulation	AMPK–JNK, autophagy, Nrf2, anti-apoptotic signaling	Improved hippocampal memory, neurogenesis, synaptogenesis; improved global cognition; reduced amyloid/tau burden [105]	High: counteracts oxidative stress and inflammation underlying chemobrain
Resveratrol [92]	Paclitaxel neurotoxicity models	Weak ERα/ERβ agonist	SIRT1–PGC-1α, M2 microglial polarization	Improved spatial learning; reduced neuronal apoptosis and oxidative stress	Supports mitochondrial and microglial resilience during chemotherapy
Genistein [106]	Clinical trial (prodromal AD)	Preferential ERβ agonist	ERβ–CREB, PI3K/Akt, epigenetic modulation	Improved verbal learning and visuospatial memory; stabilized amyloid uptake	Highly relevant: ERβ-rich brain regions; estrogen deficiency
Daidzein [31,111]	Preclinical and mechanistic studies	ERβ agonist	Antioxidant, anti-inflammatory cascades	Cognitive resilience; reduced oxidative stress	Menopause-associated cognitive decline
Coumestrol [31,111]	Receptor-binding and signaling studies	High-affinity ERα/ERβ ligand	MAPK/ERK, PKC	Strong estrogenic neuro-signaling	Potent phytoestrogen in low-estrogen states
Oleuropein aglycone (OleA) [18,107,119]	Neuroinflammation & CICI models	Indirect ER/GPER signaling	COX-1/2 inhibition, TREM2 modulation, autophagy	Reduced cytokines (IL-6, TNF-α); improved redox balance	Limits chemotherapy-induced neuroinflammation
Hydroxytyrosol (HT) [18,107,113,119]	Neuronal and AD models	ERβ upregulation; GPER inverse agonist	AMPK–SIRT1–PGC-1α, ERK1/2	Enhanced mitochondrial efficiency; reduced ROS; cognitive improvement	Mimics estrogenic neuroprotection in menopause
Mangiferin/Morin [120]	Excitotoxicity & ischemia models	ER-independent	Mitochondrial rescue, calpain inhibition	Reduced neuronal death; anti-apoptotic effects	Protects against metabolic and oxidative injury

## Data Availability

No new data were created or analyzed in this study. Data sharing is not applicable to this article.
